# Book Reviews

**Published:** 1911-10

**Authors:** 


### BOOK REVIEWS

A MANUAL OF DISEASES OF INFANTS AND CHILDREN. By John Ruhrah, Clinical Professor of Diseases of Children, College of Physicians and Surgeons, Baltimore. Third Revised Edition. 12mo. volume of 534 pages, fully illustrated. Philadelphia and London: W. B. Saunders Company, 1911. Flexible leather, $2.50 net.

This little book has been prepared for the medical student, not to supplant the larger text-book, but to enable the student to grasp quickly the more important part of pediatrics, and has proven so useful to the general practitioner as well, that the third edition has now been necessary.

---

A MANUAL OF PRACTICAL HYGIENE for Students, Physicians and Health Officers. By Chas. Harrington, M. D., Late Professor of Hygiene in the Harvard Medical School. 4th Revised and Enlarged Edition, by M. W. Richardson, M. D., Sec. to the State Board of Health of Massachusetts. 850 pages, with many illustrations. Lea & Febiga. Philadephia.

The splendid work is without doubt the best of its kind in the English language.

DISEASES OF THE STOMACH AND UPPER ALIMENTARY TRACT. By Anthony Bassler, M. D., Visiting Gastroenterologist to the Peoples' Hospital, and Vis'ting Physician to the St. Mark's Hospital Clinic; Member of the American Gastroenterological and American Medical Associations; Fellow of the New York Academy of Medic'ne, etc.

During the last few years the field of medical literature has been simply inundated with books on diseases of digestion, some of them good, some mediocre, some "a weariness to the flesh."

This work by our genial friend Anthony beolngs to the first class, and in addition possesses the merit of being couched in nice English, showing a decent respect for the gentle art of rhetoric.

Since the appearance of Watson's Practice of Physic, whose pages blossomed with beautifuly expressed scientific thoughts; and Meigs' practice of Midwifery, wherein the art-obstetrical was depicted in glowing word picture, little attention was paid to the "classy" literary style in medical writings, until Dr. Weir Mitchell awoke the echoes, whilee Dr. Osler came in a close second.

We note, therefore, with peasur the advent of a new contributor in ths field, who gives us a well-considered, erudite exposition of the present views of gastroenterology, and fails not to clothe his thoughts in pleasing garments.

This volume is well worth a place in the library of every progressive physician, while the publishers, F. A. Davis Co., of Philadelphia, have done their part most creditably.

G. M. N.

---

A MANUAL OF MATERIA MEDICA. For Medical Students. By E. Quin Thornton, M. D., Asst. Prof. Materia Medica in the Jeqerson Medical College, Philadelphia. Published by Lea & Febiger, Phila.

The writer has presented clearly the facts necessary for the foundation of the course in therapeutics so far as the names, sources, physical properties, composition, doses and methods of administration of drugs and preparations are concerned. This book will prove of great value both to the student and physician.

---

THE PATHOLOGY OF THE LIVING AND OTHER ESSALS. By B. G. A. Moynihan, M. S. (London), F. R. C. S.; Honorary Surgeon to Leeds General Infirmary; Professor of Clinical Surgery at the Uniersity ofv Leeds, England. 12mo of 260 pages. Philadelphia and London: W. B. Saunders Company, 1910. Cloth, $2.09 net.

Moynihan presents some new views concerning mony problems which are at the present engaging the attention of surgeons. He urges the importance of knowing the pathology of condition during life rather than after post-mortem changes have taken place.

### OUR OVERCROWDED PROFESSION.

Our friends, the lawyers of this judicial district, have found it necessary to clean house; out of 800 candidates only 138 men passed a recent examination. Each of the 138, before he can practice, must submit the most unimpeachable evidence of good moral character; and it is likely that in the near future the examinations, both preliminary and final, will be much more severe than heretofore. Justices of the Supreme Court, sitting in the Trial and Special Terms in this county, have complained of the lack of legal learning shown by many lawyers; and councilors listening to these incompetents have expressed surprise at their ability to pass examinations.

An incompetent physician, or one of poor moral fibre, is a greater menace to the race than a poor lawyer; and, with all deference to members of sister professions, we state our belief that the physician moves upon a higher ethical plane, theoretically, than theirs. Yet "fee splitting" and other practices of the kind have become so notorious of late years throughout the land as to force mention from a president of the American Medical Association and, in one county, to compel investigation by the Board of Regents of this State.

The formerly high tone of the profession is lowered by the crowding into its ranks of persons of inferior mental and moral integrity, and for this we can blame only the multiplicity of medical schools, founded, many of them, solely to advertise the organizing body and guaranteeing implicitly his degree to each matriculate who keeps up his fees. In a posthumous article by Dr. Frank P. Foster, published in our issue of August 26th, it was stated that it is hopeless to expect the schools of New York to approach the excellence of the continental European establishments owing to the number of the former. New York has five large medical schools, while a European metropolis is content with one. The competition which is said to be an excellent thing in commerce, should have no part in the training of men to fill the most exacting and the most honorable of professions. Concentration of intellect and energy into one powerful source is the ideal. One school would be sufficient in this city to accommodate all the men who were able to qualify under the best conditions, viz., a carefully devised education grafted upon a moral type of exceptional excellence.

Some of the schools now demand an academic degree of matriculates; this is something, but not yet enough. The preliminary education should comprise biology, zoology, botany, and, above all, advanced chemistry and some practice with the microscope. Latin and Greek, indeed, give mental training and help in the understanding of the nomenclature, but French and German might well replace them, if thoroughly taught. What is required is the acquisition of the scientific type of mind; the mind that accepts nothing from authority, but approaches every problem as a skeptic and yields only to irrefragable proof. There should be, moreover, some way to impress upon the student the really great dignity, aye, sacredness of the medical profession, to whose keeping is intrusted not only life, but happiness in life.

As Europe has found five years to be required for the proper training of the physician, so must we find them necessary. There should be still more personal contact between instructor and pupil and there are other things to be communicated beside scientific knowledge. A wrong type of instructor must have handled many of the present members of the profession. A fee splitter has lost some of the fine edge of honor that belongs to a gentleman and scholar, or he has never had it conferred upon him. Such a man degrades medicine to a trade. Furthermore, there are physicians who are mere checkers of symptoms; there are surgeons who are little more than expert mechanics, unable to make a diagnosis save by an "exploratory incision." There are men who when called to a patient actually ask him which "school" of medicine he believes in and offer to treat him according to his creed. There is a great multitude that never think for themselves, but must wait till some medical oracle goes into an ecstacy.

The gate to be shut upon men of these types is not that of exit, but the portal of entry. If the doctor is to be of high mental and moral excellence, so must the student be. The example of the lawyers is to be highly commended; we must be severe in our entrance examinations and exigent in our moral demands of the candidate. Character is wanted in the medical as in the legal profession. Abuse, misunderstanding, misrepresentation, hardship are part of the game; it is the renegade who dodges them by taking refuge in quackery. There is not room for the man of low ideals and preliminary education is the best means of extirpating him. A certain fine quality of mind is wanted of the aspirant to medical honors, a quality that has hopes of an old-fashioned kind, of distinction through means other than material success. If the latter comes well and good; but it is not the chief end in view. The doctor must love his neighbor and his end is service.

We do not think we have indicated a type of being more than human; there is a grand minority of the profession who embody all the excellences we wish to see permeate the whole. Such men were probably always superior to the rank and file. There are more of them coming to maturity and they should be seized upon for the profession to the exclusion of the less worthy. A certain smart and advertising kind of man, who too often typifies the profession to outsiders, is precisely the kind we wish to exclude. Several mental and moral tests will accomplish his exclusion. We do not like to hear our prominent men spoken of, as they now are, in a tone of amused contempt by convalescents who somehow feel that they have been bested as well as healed. There is no hope for profession or public unless the lines are rigidly drawn. We want none but high priests behind the veil of the temple.—Ed. N. Y. Med. Jour.

---

### HEATING AND VENTILATION.

It has long been the custom of hygienists to insist that ventilation or perflation of crowded rooms is necessary in order to remove poisonous emanations arising from the human body. More than twenty years ago, however, observations made to determine the nature of the alleged poisonous properties of the expired air and of "crowd poison" led to negative results. No toxic organic constitutent could be demonstrated in the air of crowded rooms, and as for the carbon dioxid, it was so relatively slight in amount that it could not be held responsible for the physiological effects observed, and could at most be regarded as "a measure of danger." To what, then are due the disagreeable consequences of remaining in an overcrowded, poorly ventilated room? There can be no doubt that such consequences are real and not imaginary, and that they range all the way from slight depression and headache to severe nausea, vomiting and collapse.

The answer to a question that has caused much perplexity seems to be given by various physiologic experiments of the last few years. To put it briefly, the whole trouble seems to be due to a disturbance of the normal thermal relations of the body. It is a common observation that the depression and fatigue experienced on a hot, humid August day are very similar to the feelings that develop in a crowded "close" room. The cause is apparently the same in both cases, namely, interference with the normal rate of loss of body heat. At least three atmospheric factors may be concerned in such interference: high temperature of the ambient air, high moisture content and lack of air movement. Paul kept healthy persons for several hours in a close cabinet until the carbon dioxid rose to 100 or 150 parts per ten thousand—more than ten times the amount usually stated as "allowable"—but so long as the temperature and moisture element were kept low no symptoms of illness or discomfort developed. Other experimenters have reached the same result by simply having electric fans whirled in such an experimental cabinet. The motion these imparted to the air was sufficient to facilitate a normal, physiologic loss of heat from the body in spite of high temperature and humidity. Similar cabinet experiments in which the subject was enabled to breathe the fresh outside air through a tube, but was otherwise subjected to the conditions of a close room, likewise showed that the symptoms attributed to "bad ventilation" are in no wise due to poisons excreted in the breath. The upshot of all such experiments is that it is not the chemical constitution of indoor air that is injurious, but the overheating, the stagnation and sometimes the moisture content.

Among the more important recent papers dealing with this question may be mentioned especially an article by Crowder on the ventilation of sleeping-cars. In addition to an admirable critical summary of the experimental evidence on the subject, the article contains valuable data from the writer's own study of car ventilation. Much of the discomfort experienced by sleeping-car travelers is undoubtedly due, as Crowder suggests, to overheating and not to insufficient air-supply. Important practical results may be looked for in the field of ventilation as soon as engineers come to realize and apply the knowledge gained by hygienists in the past few years. We are likely to hear less about "impure air" and more about stagnation and overheating.

All this does not, of course, mean that there is no harm in crowded rooms or that the open-air treatment of tuberculosis is based on false assumptions. The investigations cited merely substitute the right for the wrong impression of observed facts. Bodily resistance, as bacteriologists have shown us, may be lowered by a variety of factors. It might conceivably be diminished by the inhalation of "crowd poison," or by interference with the heat regulation of the body. The latter seems to be the true explanation of the undoubtedly injurious effect of overcrowded rooms. So far as tuberculosis is concerned, the predisposing effect of damp houses and damp climates is well known. The experiments recorded by the investigators mentioned above suggest that there are yet undeveloped possibilities in the control of indoor climate. Precise knowledge of the factors concerned in producing the bad effects of overcrowding is the first step toward a scientific remedy for the condition.

